# Toll-Like Receptor 4 in Paraventricular Nucleus Mediates Visceral Hypersensitivity Induced by Maternal Separation

**DOI:** 10.3389/fphar.2017.00309

**Published:** 2017-05-29

**Authors:** Hui-Li Tang, Gongliang Zhang, Ning-Ning Ji, Lei Du, Bin-Bin Chen, Rong Hua, Yong-Mei Zhang

**Affiliations:** ^1^Jiangsu Province Key Laboratory of Anesthesiology, Xuzhou Medical UniversityXuzhou, China; ^2^Department of Anesthesiology, The First Affiliated Hospital of Soochow UniversitySuzhou, China; ^3^Department of Pharmacology, College of Basic Medical Sciences, Anhui Medical UniversityHefei, China; ^4^Department of Emergency, Affiliated Hospital of Xuzhou Medical UniversityXuzhou, China

**Keywords:** neonatal maternal separation, visceral hypersensitivity, PVN, TLR4, CRF

## Abstract

Neonatal maternal separation (MS) is a major early life stress that increases the risk of emotional disorders, visceral pain perception and other brain dysfunction. Elevation of toll-like receptor 4 (TLR4) signaling in the paraventricular nucleus (PVN) precipitates early life colorectal distension (CRD)-induced visceral hypersensitivity and pain in adulthood. The present study aimed to investigate the role of TLR4 signaling in the pathogenesis of postnatal MS-induced visceral hypersensitivity and pain during adulthood. The TLR4 gene was selectively knocked out in C57BL/10ScSn mice (*Tlr4*^-/-^). MS was developed by housing the offspring alone for 6 h daily from postnatal day 2 to day 15. Visceral hypersensitivity and pain were assessed in adulthood. *Tlr4*^+/+^, but not *Tlr4*^-/-^, mice that had experienced neonatal MS showed chronic visceral hypersensitivity and pain. TLR4 immunoreactivity was observed predominately in microglia in the PVN, and MS was associated with an increase in the expression of protein and/or mRNA levels of TLR4, corticotropin-releasing factor (CRF), CRF receptor 1 (CRFR1), tumor necrosis factor-α, and interleukin-1β in *Tlr4*^+/+^ mice. These alterations were not observed in *Tlr4*^-/-^ mice. Local administration of lipopolysaccharide, a TLR4 agonist, into the lateral cerebral ventricle elicited visceral hypersensitivity and TLR4 mRNA expression in the PVN, which could be prevented by NBI-35965, an antagonist to CRFR1. The present results indicate that neonatal MS induces a sensitization and upregulation of microglial TLR4 signaling activity, which facilitates the neighboring CRF neuronal activity and, eventually, precipitates visceral hypersensitivity in adulthood.

**Highlights**

(1) Neonatal MS does not induce chronic visceral hypersensitivity and pain in *Tlr4*^-/-^ mice.
(2) Neonatal MS increases the expression of TLR4 mRNA, CRF protein and mRNA, CRFR1 protein, TNF-α protein, and IL-1β protein in *Tlr4*^+/+^ mice.
(3) TLR4 agonist LPS (i.c.v.) elicits visceral hypersensitivity and TLR4 mRNA expression in the PVN.

## Introduction

Neonatal maternal separation (MS) and other adverse events in early life alter neural plasticity and the developmental trajectory across the nervous system (Hulshof et al., 2011; Ji et al., 2014; Wang et al., 2015). Visceral hypersensitivity is common characteristic in subjects suffering from irritable bowel syndrome (IBS) and other functional gastrointestinal disorders (Chey et al., 2015). In the mice, neonatal MS increases the susceptibility of visceral hypersensitivity and pain in adulthood (Moloney et al., 2012). The cause of visceral hypersensitivity is multifactorial and is believed to be triggered by environmental stressors and/or psychological factors, as well as neuroinflammation (Coutinho et al., 2002; Chung et al., 2007). Recent studies have shown that toll-like receptor 4 (TLR4)-related neuroinflammation is a critical factor for early life stress-induced adult visceral hypersensitivity (Chen et al., 2015). TLR4s in the central nervous system are expressed predominately in glia (Lee et al., 2013). Activation of TLR4 increases the production and secretion of cytokines interleukin-1β (IL-1β), tumor necrosis factor-α (TNF-α) and other proinflammatory factors via the myeloid differentiation factor 88 (MyD88)-dependent signaling cascade (O’Neill, 2008). As a part of the outer membrane of Gram-negative bacteria, lipopolysaccharide (LPS) has been shown to initiate multiple intracellular signaling events and inflammatory responses by activating TLR4 (Schletter et al., 1995; Park et al., 2009). We recently demonstrated that neonatal colorectal distension (CRD), a physical stress in early life, increases sensitization of the TLR4/MyD88 signaling pathway in the paraventricular nucleus (PVN) and in visceral pain precipitation in rats (Chen et al., 2015). To date, the effect of TLR4 signaling in MS-induced visceral hypersensitivity has not been explored.

The hypothalamic PVN collects multiple sources of afferents to generate autonomic outputs for pain modulation. Corticotropin-releasing factor (CRF) neurons in PVN augment adrenocorticotropic hormone (ACTH) and corticosteroid levels via the HPA axis (de Kloet et al., 2005; Bravo et al., 2011). Early life stress can disrupt PVN neuroplasticity and the HPA axis (Van den Bergh et al., 2008; Green et al., 2011; Amath et al., 2012). MS evokes an exaggerated increase in blood ACTH, plasma corticosterone, and CRF levels in the amygdala, locus coeruleus, and hypothalamus in adulthood (Plotsky and Meaney, 1993; Suchecki et al., 1993, 1995). We found that CRD induces visceral hypersensitivity and elevations in plasma cortisol levels and CRF protein expression in the PVN (Yu et al., 2014; Chen et al., 2015). CRF and the HPA neuraxis modulate neuropathic pain (Fu and Neugebauer, 2008) and analgesia (Lariviere and Melzack, 2000), and the PVN may be a pivotal region modulating visceral hypersensitivity. CRF increases TLR4 expression via corticotropin-releasing factor receptor 1 (CRFR1) (Chaniotou et al., 2010). However, the machinery for TLR4 signaling in the PVN on the pathogenesis of MS-induced visceral hypersensitivity and pain remain elusive.

A feasible method for examining the neurobiology of MS is through the use of a rodent model of MS in which pups are deprived of their dams (Hall, 1998). In the present study, we used wild-type and TLR4 gene knockout mice to explore the TLR4 signaling pathway and neuroinflammation on MS-induced visceral hypersensitivity and pain. Our results revealed for the first time that MS-associated visceral hypersensitivity was related to an increase in TLR4 signaling and neuroinflammation in the PVN.

## Materials and Methods

### Animals

C57BL/10JNju (*Tlr4*^+/+^) and C57BL10/ScNJNju (*Tlr*4^-/-^) mice came from the Model Animal Research Center of Nanjing University (Nanjing, China). Wild-type and TLR4 gene knockout mice used in this experiment were bred in our laboratory to produce the offspring. Mice were group housed in a constant temperature and humidity (22°C and 50%, respectively) colony maintained on a 12-h light-dark cycle (lights on at 07:00 am) and *ad libitum* access to food and water. All procedures were conducted in accordance with the guidelines described in the National Institutes of Health’s *Guide for the Care and Use of Laboratory Animals* (NIH Publication No. 8023, revised 1978) and the International Association for the Study of Pain, and were approved by the Institutional Animal Care and Use Committee at Xuzhou Medical College.

### Mouse Model of Maternal Separation

The MS protocol was conducted as previously described in detail (Leon Rodriguez and Duenas, 2013; Wang et al., 2016). Briefly, male and female mice in both the *Tlr4*^+/+^ and *Tlr4*^-/-^ strains were mated for litters. After birth, all pups were randomly divided into the MS group and the non-maternal separation (non-MS) group. Neonatal mice were removed from their littermates and dams for 6 h/day (8:00–11:00 am and 2:00–5:00 pm) from postnatal day 2 to day 15 before being returned to standard housing. During the separation, both dams and newborns were housed in the same room, but with different cages. The pups in the non-MS group remained in their home cages with dams and siblings. Tests were conducted when mice reached 2 months of age and weighed 20–25 g.

### Assessment of Abdominal Withdrawal Reflex (AWR) and Pain Threshold

Mice were fasted for 18 h with water *ad libitum* before testing. A custom-designed balloon (1 cm in diameter and 2 cm in length), connected to a syringe for inflating the balloon via a tube, was inserted into the colorectal intestine 0.5 cm above the anus under isoflurane anesthesia. Mice were placed in small Lucite cubicles (20 cm × 20 cm × 9 cm) and allowed to habituate to them for 15 min. During the Abdominal Withdrawal Reflex (AWR) score tests, graded distension was produced by rapidly inflating the balloon to a desired pressure (20, 40, 60, or 80 mmHg) for 20 s followed by a 4-min break. The AWR was scored as: 0, no behavioral response to distension; 1, immobility or head movement; 2, light abdominal wall contraction but no lifting from the table; 3, obvious abdominal wall contraction and lifting from the table; 4, concave arching of the back or lifting of the pelvis (Zhang et al., 2016b). The distension pain threshold was defined by a stimulus intensity that evoked a visible contraction of the abdominal wall or an AWR score of 3. During pain threshold testing, CRD was applied in an increment of 10 mmHg starting at 10 mmHg for 20 s followed by a 4-min break. The distension at each condition was performed in triplicate and the results were averaged for further analysis.

### Immunofluorescence Labeling

After deeply anesthesia, the mice were perfused transcardially with 20 mL of 0.9% saline, followed by 20 ml 4% paraformaldehyde. Brains were harvested and further fixed in 4% paraformaldehyde solution at 4°C overnight before being equilibrated in a 30% sucrose solution. The PVN region was cut in 35-μm thickness slices with a cryostat (Leica CM1800; Heidelberg, Germany). Selected sections were washed with 0.01 M PBS and incubated with 10% donkey serum in PBS containing 0.3% Triton X-100 for 2 h at room temperature before being incubated with goat anti-CRF antibody (sc-1759; 1:50; Santa Cruz, CA, United States), goat anti-CRFR1 antibody (sc-12383; 1:50; Santa Cruz, CA, United States), a mixture of mouse anti-TLR4 (ab-22048; 1:100; Abcam, United Kingdom) and rabbit anti-Iba-1 (019-19741; 1:300; Wako, Japan) antibodies, or a mixture of rabbit anti-TLR4 (ab13556; 1:200; Abcam, United Kingdom) and mouse anti-GFAP (#3670; 1:300; Cell Signaling, Danvers, MA, United States) antibodies for 48 h at 4°C. After TBS-T washing, Alexa 488 donkey anti-rabbit IgG (1:200), Alexa 594 donkey anti-mouse IgG (1:200), or Alexa 594 donkey anti-goat IgG (1:200) was added to the corresponding sections and incubated for 2 h at room temperature. Tissue sections were mounted with 50% glycerol mounting medium. The PVN was visualized with a confocal laser scanning microscope (FV1000; Olympus, Tokyo, Japan). Tissue images were processed using Image Pro-Plus 5.0 software (Media Cybernetics, Silver Spring, MD, United States) for cell counting.

### Microinjection of LPS and NBI-35965 in the Lateral Ventricle (LV)

Under deep isoflurane anesthesia, the head of each mouse was secured in a stereotaxic frame with the skull in a horizontal flat position. A Hamilton microsyringe was insert into the right Lateral Ventricle (LV; A/P, -0.56 mm; M/L, ±1.0 mm; D/V, -1.5 mm from bregma) (Franklin and Paxinos, 2008) via a hole drilled into the skull. LPS (0.5 μL of 1 ng/μL) was infused into the LV over 1 min, and the needle was kept in place for another 3 min to allow the drug to distribute evenly. Mice were returned to their cages, and behavioral tests were conducted 2 h later. In another cohort of mice, 1 μL of the CRFR1 antagonist NBI-35965 (1 μg/μL) was infused into the LV 45 min before the LPS administration. The control mice received the same surgical treatment except that the drug was replaced with the same volume of sterile solvent. Mice were sacrificed immediately after the behavioral test, and the PVN regions were collected for subsequent Enzyme-Linked Immunosorbent Assay (ELISA) and Reverse Transcription-Polymerase Chain Reaction (RT-PCR) tests. Isofluorane anesthesia itself did not affect mouse behaviors (Supplementary Figure S1).

### Enzyme-Linked Immunosorbent Assay (ELISA)

The levels of IL-1β, IL-6, and TNF-α were quantified using corresponding ELISAs. Briefly, the PVN was collected and homogenized in a RIPA lysis buffer (P0013B, Beyotime, China). The homogenates were centrifuged at 12,000 ×*g* (4°C) for 15 min. The supernatants were collected and assayed in duplicates with IL-1β, IL-6, and TNF-α assay kits (ExCell, China) according to manufacturer’s instructions. Cytokine concentrations are expressed as pg/mg protein.

### Reverse Transcription-Polymerase Chain Reaction (RT-PCR)

The PVN mRNA levels of TLR4, MyD88, and CRF were measured by PCR. Total RNA was isolated from PVN tissues using an RNA extraction kit (DP431, Tiangen, China). The RNA level was measured by spectrophotometric analysis (OD260/280). RNA was transcribed to cDNA using M-MLV Reverse Transcriptase (D263915) and dT primers. PCR amplification was conducted with Taq polymerase using 40 cycles at 94°C for 30 s, 58°C for 30 s, and 72°C for 1 min. The primers for TLR4 were 5′-AAACTCAGCAAAGTCCCTGATGAC-3′ (sense), 5′-CGTA GAAACTGTAAGTCGTTGACAG-3′ (antisense); for MyD88 were 5′-ATGGTGGTGGTTGTTTCTGACGA-3′ (sense), 5′-GCAAGGGTTGGTATAGTCGCATATA-3′ (antisense); for CRF were 5′-CGCAGCCGTTGAATTTCTTG-3′ (sense), 5′-GCAG CGGGACTTCTG-3′ (antisense); and for GAPDH were 5′-AGGCCGGTGCTGAGTATGTC-3′ (sense), 5′-TGCCTGCTTC ACCACCTTCT-3′ (antisense). The PCR products were electrophoresed using 1.5% agarose gels in TAE buffer at 140 V for approximately 30 min. The gels were imaged with the Bio-Rad Gel Doc XR imaging system, and pictures were acquired with a Cannon camera and analyzed with ImageJ software (NIH, Bethesda, MD, United States).

### Statistical Analysis

Data are expressed as means ± SEM. The time course for the number of abdominal contractions was analyzed using a two-way repeated measures ANOVA. Student’s *t*-test, one-way ANOVA, and two-way ANOVA were conducted where indicated. If a statistical significance was found, *post hoc* Bonferroni or Tukey’s multiple comparisons tests were applied. *p* < 0.05 considered statistically significant.

## Results

### Adult *Tlr4*^+/+^ Mice Subjected to Neonatal MS Show Increased Visceral Hypersensitivity

**FIGURE 1:**
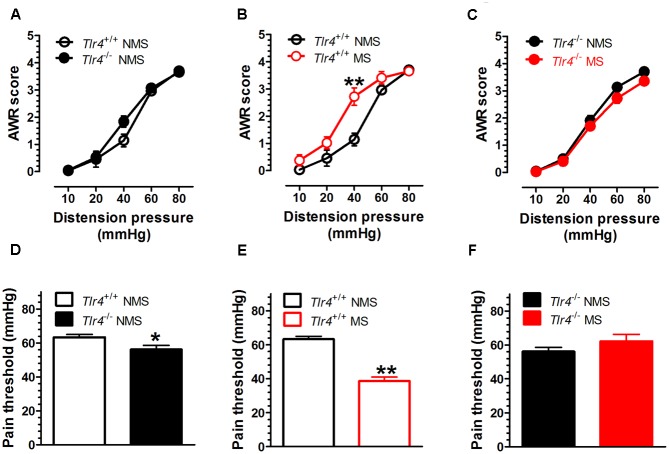
**Visceral hypersensitivity is increased in adult *Tlr4*^+/+^ mice subjected to neonatal MS.** Neonatal mice were subjected to MS, and their behavioral assessment was conducted during adulthood. **(A)** There was no difference in AWR scores between *Tlr4*^+/+^ (*n* = 9) and *Tlr4*^-/-^ mice (*n* = 11) that did not experience MS (non-MS, NMS). **(B)** AWR scores for *Tlr4*^+/+^ mice that underwent MS were increased compared with those for non-MS *Tlr4*^+/+^ mice (*n* = 9 in each group; *p* < 0.01). **(C)** By contrast, MS *Tlr4*^-/-^ mice (*n* = 10) showed decreased AWR scores compared with non-MS *Tlr4*^-/-^ mice (*n* = 13; *p* < 0.05). **(D)** The pain threshold in *Tlr*4^-/-^ mice (*n* = 13) was decreased compared with that in *Tlr*4^+/+^ mice (*n* = 13; *p* < 0.05). **(E)** In *Tlr4*^+/+^ mice, MS (*n* = 8) induced a decrease in the pain threshold compared with that in non-MS mice (*n* = 9). **(F)** However, MS (*n* = 9) did not alter the pain threshold in non-MS *Tlr*4^-/-^ (*n* = 13) mice (*p* > 0.05). ^∗^*p* < 0.05, ^∗∗^*p* < 0.01. Data are expressed as means ± SEM.

The TLR4 gene knockout did not alter the AWR score induced by CRD in the non-MS group of mice. A two-way repeated measures ANOVA on AWR scores revealed a significant main effect of distension pressure [*F*_(4,72)_ = 217.06, *p* < 0.001], but did not reveal significant effects of group [*F*_(1,18)_ = 2.22, *p* = 0.154] and the interaction between group and distension pressure [*F*_(4,72)_ = 1.97, *p* = 0.108] (**Figure 1A**). The *Tlr4*^+/+^ mice subjected to MS had increased AWR scores compared with those for non-MS *Tlr4*^+/+^ mice. A two-way repeated measures ANOVA on AWR scores revealed significant main effects of group [*F*_(1,16)_ = 2.22, *p* = 0.005], distension pressure [*F*_(4,64)_ = 130.32, *p* < 0.001], and the interaction between group and distension pressure [*F*_(4,64)_ = 5.27, *p* = 0.001; **Figure 1B**]. By contrast, the *Tlr4*^-/-^ mice subjected to MS presented decreased AWR scores compared with those for non-MS *Tlr4*^-/-^ mice. A two-way repeated measures ANOVA on AWR scores revealed significant main effects of group [*F*_(1,21)_ = 4.49, *p* = 0.046] and distension pressure [*F*_(4,84)_ = 290.85, *p* < 0.001], but not for the interaction between group and distension pressure [*F*_(4,84)_ = 0.86, *p* = 0.489; **Figure 1C**). *Tlr4*^-/-^ mice showed a decrease in pain threshold compared with that for *Tlr*4^+/+^ mice [*t*(20) = 2.23, *p* = 0.038; **Figure 1D**). In wild-type mice, MS induced a decrease in pain threshold compared with that in non-MS mice [*t*(15) = 8.87, *p* < 0.001; **Figure 1E**]. In *Tlr*4^-/-^ mice, MS did not alter the pain threshold compared with that in non-MS mice [*t*(20) = 1.38, *p* = 0.18; **Figure 1F**].

### Adult Mice Subjected to Neonatal MS Show Increased PVN TLR4 Expression

**FIGURE 2:**
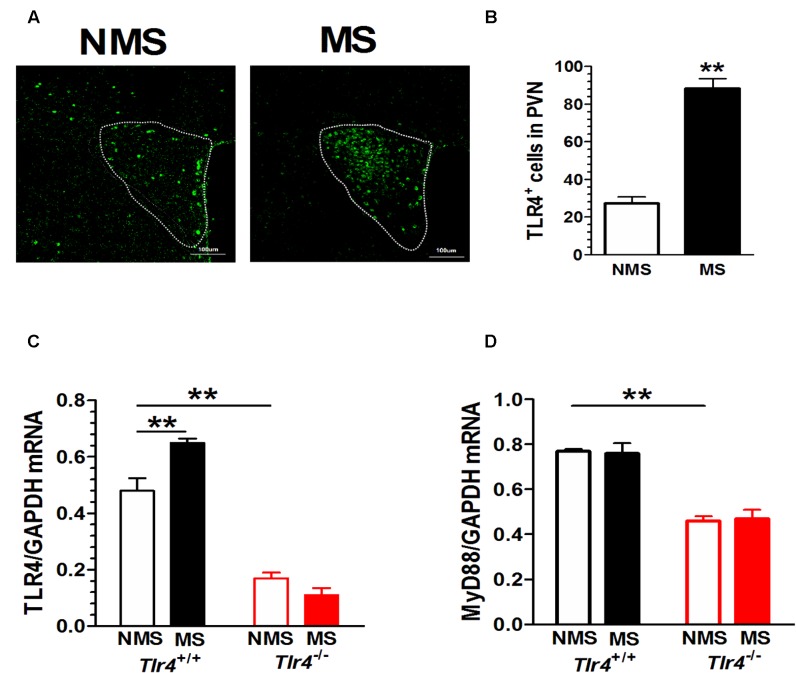
**PVN TLR4 expression is increased in adult mice that had experienced neonatal MS.** Mice that had experienced neonatal MS display a significant increase in **(A)** the expression of PVN TLR4^+^ immunostaining and in **(B)** the number of TLR4-positive cells in the PVN compared with mice that had not experienced MS (88.20 ± 5.38 vs. 27.20 ± 3.53; *p* < 0.01; *n* = 10 in each group). **(C)** Compared with *Tlr4*^+/+^ mice, *Tlr4*^-/-^ mice displayed significantly lower TLR4 expression (*p* < 0.001). Compared with non-MS, MS was associated with a significant increase in TLR4 mRNA expression in *Tlr4*^+/+^ but not in *Tlr4*^-/-^ mice (*n* = 4 in each group). **(D)** *Tlr4*^-/-^ mice displayed significantly lower expression of MyD88 mRNA than *Tlr4*^+/+^ mice do (*n* = 4 in each group; *p* < 0.001). ^∗∗^*p* < 0.01. Data are expressed as means ± SEM.

Compared with mice in the non-MS group, adult mice that experienced neonatal MS showed a significant increase in TLR4^+^ immunostaining in the PVN (**Figure 2A**). The numbers of TLR4^+^ cells in the PVN of mice in the non-MS and MS groups were 27.20 ± 3.53 and 88.20 ± 5.38, respectively [*t*(18) = 9.49, *p* < 0.001; **Figure 2B**]. TLR4 gene knockout altered the TLR4 mRNA level. A two-way ANOVA on TLR4 mRNA levels in the PVN revealed a significant main effect of genotype [*F*_(1,12)_ = 202.93, *p* < 0.001] and the interaction between genotype and MS [*F*_(1,12)_ = 15.68, *p* = 0.002], but no effect of MS [*F*_(1,12)_ = 3.97, *p* = 0.070]. *Tlr4*^-/-^ mice presented a significantly lower expression of TLR4 mRNA than *Tlr4*^+/+^ mice did. Compared with non-MS, MS was associated with a significant increase in TLR4 mRNA expression in *Tlr4*^+/+^ mice (**Figure 2C**). TLR4 gene knockout significantly altered the MyD88 mRNA level. A two-way ANOVA on the TLR4 mRNA level in the PVN revealed a significant main effect of genotype [*F*_(1,12)_ = 91.12, *p* < 0.001], but no effect of MS [*F*_(1,12)_ = 0.007, *p* = 0.936] and the interaction between genotype and MS [*F*_(1,12)_ = 0.159, *p* = 0.697]. The expression of MyD88 was significantly lower in *Tlr4*^-/-^ than that in *Tlr4*^+/+^ mice (**Figure 2D**).

**FIGURE 3:**
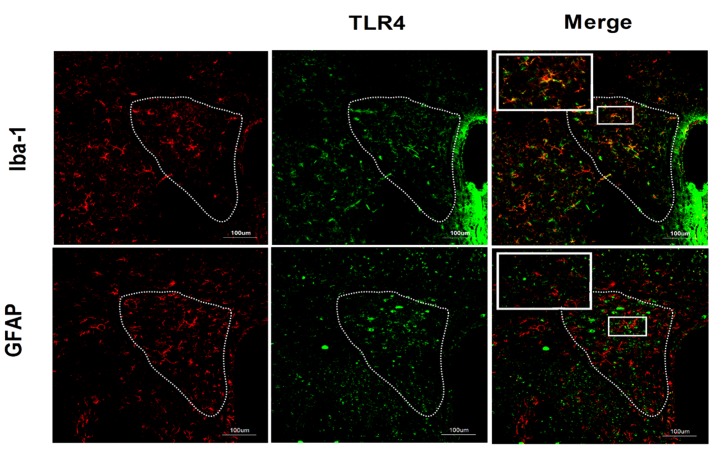
**Co-expression of TLR4 and Iba-1 in the PVN.** Double-labeling studies showed that TLR4 was mainly co-distributed with Iba-1, a microglia marker. There was little co-labeling of TLR4 and GFAP, an astrocyte marker. Scale bar = 100 μm.

The results of double-labeling studies showed that TLR4 mainly co-distributed with Iba-1, a microglia marker. There was little co-labeling of TLR4 and GFAP, an astrocyte marker (**Figure 3**).

### Adult Mice Subjected to Neonatal MS Show Increased CRF and CRFR1 Expression

**FIGURE 4:**
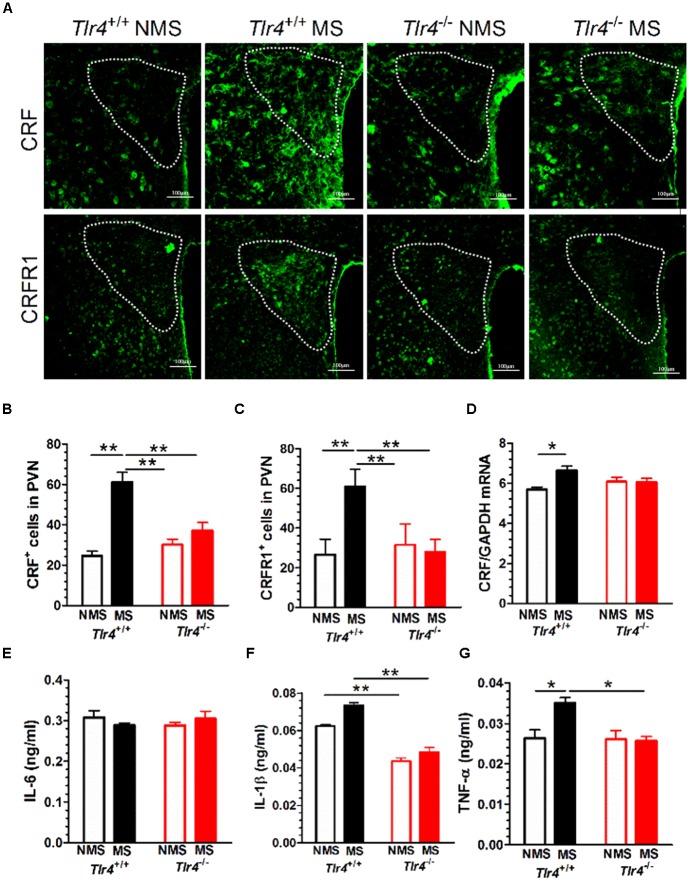
**Adult mice subjected to neonatal MS show increased expression of CRF, CRFR1, IL-1β, and TNF-α. (A)** The expression of CRF and CRFR1 immunofluorescence in the PVN of MS *Tlr4*^+/+^ mice was significantly increased compared with that in non-MS *Tlr4*^+/+^mice. A similar trend was not observed in *Tlr4*^-/-^ mice. **(B)** Mice in the MS group showed a significant increase in the number of CRF^+^ cells compared with those in the non-MS group for *Tlr4*^+/+^ but not *Tlr4*^-/-^ mice (*n* = 6–8 in each group). **(C)** Mice in the MS group also showed a significant increase in the number of CRFR1^+^ cells compared with those in the non-MS group for *Tlr4*^+/+^ but not *Tlr4*^-/-^ mice (*n* = 6–9 in each group). **(D)** Similarly, the MS group displayed a significant increase in CRF mRNA expression compared with the non-MS group for *Tlr4*^+/+^ but not *Tlr4*^-/-^ mice (*n* = 4 in each group). **(E)** Although MS did not affect IL-6 expression (*n* = 6 in each group), **(F)** *Tlr4*^+/+^ mice in the MS group showed an increase in IL-1β expression compared with those in the non-MS group (*n* = 3–5 in each group). **(G)** The MS group was associated with a significant increase in TNF-α expression compared with the non-MS group for *Tlr4*^+/+^, but not for *Tlr4*^-/-^, mice (*n* = 6 in each group). ^∗^*p* < 0.05, ^∗∗^*p* < 0.01. Data are expressed as means ± SEM.

Immunofluorescence results revealed that *Tlr4*^+/+^ but not *Tlr4*^-/-^ mice subjected to MS displayed a significant increase in CRF and CRFR1 immunostaining (**Figure 4A**). A two-way ANOVA examining CRF^+^ cell counts in the PVN revealed significant main effects of genotype [*F*_(1,25)_ = 7.03, *p* = 0.014] and MS [*F*_(1,25)_ = 39.06, *p* < 0.001] as well as of the interaction between genotype and MS [*F*_(1,25)_ = 86.82, *p* < 0.001]. The number of CRF^+^ cells was not significantly different between *Tlr4*^+/+^ non-MS mice and *Tlr4*^-/-^ non-MS mice. However, *Tlr4*^+/+^ mice subjected to MS showed a significant increase in the number of CRF^+^ cells compared with that in non-MS *Tlr4*^+/+^ mice. There was no difference in the number of CRF^+^ cells for *Tlr4*^-/-^ mice in the MS and non-MS groups (**Figure 4B**). A two-way ANOVA on CRFR1^+^ cell counts revealed significant main effects of genotype [*F*_(1,27)_ = 21.24, *p* < 0.001], MS [*F*_(1,27)_ = 25.64, *p* < 0.001], and the interaction between genotype and MS [*F*_(1,27)_ = 39.41, *p* < 0.001]. For mice in the non-MS group, no significant difference was detected in CRFR1^+^ cells counts between *Tlr4*^+/+^ and *Tlr4*^-/-^ mice. By contrast, the CRFR1^+^ cell count was significantly higher in the MS *Tlr4*^+/+^ than that in the non-MS *Tlr4*^+/+^ mice. There was no difference in CRFR1^+^ cell counts for *Tlr4*^-/-^ mice in the MS and non-MS groups (**Figure 4C**). MS altered the CRF mRNA level. A two-way ANOVA examining CRF mRNA levels in the PVN revealed a significant main effect of MS [*F*_(1,12)_ = 6.20, *p* = 0.028] and the interaction between genotype and MS [*F*_(1,12)_ = 6.98, *p* = 0.022], but no effect of the genotype [*F*_(1,12)_ = 0.339, *p* = 0.571]. Compared with non-MS *Tlr4*^+/+^ mice, MS *Tlr4*^+/+^ mice showed an increase in CRF mRNA levels (*p* < 0.01; **Figure 4D**).

### Adult Mice Subjected to Neonatal MS Show Increased IL-1β and TNF-α, But Not IL-6, Protein Expression

Toll-like receptor 4 gene knockout did not alter the IL-6 protein level. A two-way ANOVA on IL-6 protein levels in the PVN did not revealed significant main effects of genotype [*F*_(1,15)_ = 0.915, *p* = 0.354], MS [*F*_(1,15)_ = 4.29, *p* = 0.056] and the interaction between genotype and MS [*F*_(1,15)_ = 0.509, *p* = 0.486; **Figure 4E**]. However, TLR4 gene knockout and MS altered the IL-1β mRNA level. A two-way ANOVA on the IL-1β protein level in the PVN revealed significant main effects of genotype [*F*_(1,10)_ = 74.48, *p* < 0.001] and MS [*F*_(1,10)_ = 7.32, *p* = 0.022], but no effect of the interaction between genotype and MS [*F*_(1,10)_ = 0.509, *p* = 0.486]. *Tlr4*^-/-^ mice presented a significantly lower expression of IL-1β than *Tlr4*^+/+^ mice did in both the non-MS and MS groups (*p* < 0.01; **Figure 4F**). TLR4 gene knockout and MS altered the TNF-α mRNA level. A two-way ANOVA on the TNF-α mRNA level in the PVN revealed significant main effects of genotype [*F*_(1,20)_ = 8.011, *p* = 0.010], MS [*F*_(1,20)_ = 5.92, *p* = 0.024], and significant interaction between genotype and MS [*F*_(1,20)_ = 6.954, *p* = 0.016]. Compared with the non-MS group, mice in the MS group showed an increase in TNF-α protein in *Tlr4*^+/+^ mice. There was no difference in TNF-α protein levels between the *Tlr4*^-/-^ and *Tlr4*^+/+^ mice that had experienced non-MS (**Figure 4G**).

### CRFR1 Antagonist NBI-35965 Inhibits the Visceral Hypersensitivity Induced by LPS

**FIGURE 5:**
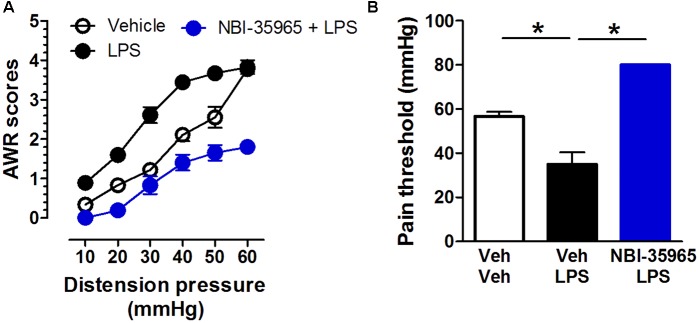
**CRFR1 antagonist NBI-35965 inhibits the visceral hypersensitivity induced by LPS. (A)** Mice received vehicle, the TLR4 agonist LPS (0.5 μg/mouse, i.c.v.), or a combination of the TLR4 antagonist NBI-35965 (10 ng/mouse, i.c.v.) and LPS. LPS induced an increase in the AWR score which could be prevented by NBI-35965. **(B)** The LPS-induced decreased in pain threshold was prevented by NBI-35965. ^∗^*p* < 0.05. Data are expressed as means ± SEM. *n* = 6 in each group.

Mice received vehicle, the TLR4 agonist LPS (0.5 μg/mouse, i.c.v.), or a combination of the CRFR1 antagonist NBI-35965 (10 ng/mouse, i.c.v.) and LPS. A two-way repeated measures ANOVA on AWR scores revealed significant main effects of time [*F*_(5,75)_ = 151.39, *p* < 0.001] and treatment [*F*_(2,15)_ = 94.46, *p* < 0.001], and significant interaction between time and treatment [*F*_(10,75)_ = 6.31, *p* < 0.001]. The *post hoc* Bonferroni multiple comparisons test showed that LPS treatment significantly increased AWR scores (vehicle vs. LPS, *p* < 0.001). NBI-35965 blocked the LPS-induced increase in AWR scores (**Figure 5A**). A one-way ANOVA examining the pain threshold results revealed a significant difference [*F*_(2,15)_ = 160.9, *p* < 0.001]. The *post hoc* Bonferroni multiple comparisons test revealed that LPS significantly decreased the pain threshold (vehicle vs. LPS). The LPS-induced decrease in pain threshold was prevented by NBI-35965 (**Figure 5B**).

### Effects of NBI-35965 on LPS-Induced Alterations in TLR4, MyD88, and CRF mRNA Levels and the Inflammatory Factors TNF-α and IL-1β

**FIGURE 6:**
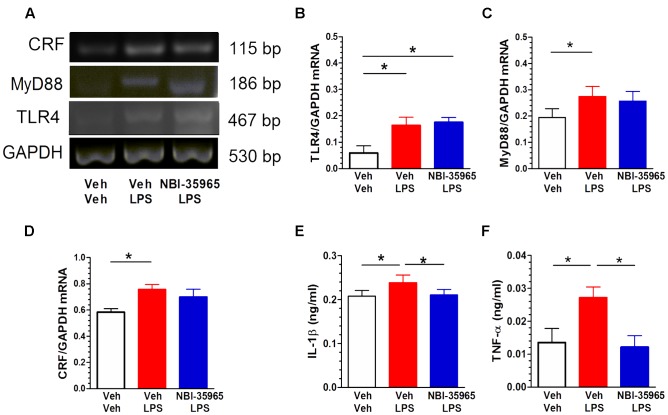
**Effects of NBI-35965 on LPS-induced alternations in TLR4, MyD88, and CRF mRNA and the levels of inflammatory factors TNF-α and IL-1β.** Mice received LPS (0.5 μg/mouse, i.c.v.). **(A)** Representative northern blots of CRF, MyD88, TLR4, and GAPDH mRNA in the PVN of mice following the administration of vehicle, LPS, or NBI-35965 + LPS (left to right lanes). **(B)** LPS increased TLR4 mRNA levels in the PVN (*n* = 4 in each group). **(C)** LPS increased MyD88 mRNA levels in the PVN (*n* = 4 in each group). **(D)** LPS increased CRF mRNA levels in the PVN (*n* = 4 in each group). **(E)** The LPS-induced increase in the PVN IL-1β level was prevented by NBI-35965 pretreatment (*n* = 6 in each group). **(F)** LPS increased the PVN TNF-α level, which could be prevented by NBI-35965 pretreatment (*n* = 4–5 in each group). ^∗^*p* < 0.05. Data are expressed as means ± SEM.

**Figure 6A** shows representative blots of CRF, MyD88, TLR4, and GAPDH mRNA levels in the PVN following treatments with vehicle, LPS, or NBI-35965 plus LPS. LPS administration (0.5 μg/mouse, i.c.v.) altered the TLR4 mRNA level in the PVN. One-way ANOVA examining TLR4 mRNA levels revealed a significant difference [*F*_(2,9)_ = 6.28, *p* = 0.02]. A *post hoc* Tukey’s multiple comparisons analysis showed that LPS increased the TLR4 level and that this increase was not blocked by NBI-35965 (**Figure 6B**). LPS also altered the PVN MyD88 mRNA level. One-way ANOVA examining MyD88 mRNA levels revealed a significant difference [*F*_(2,9)_ = 5.222, *p* = 0.03], with LPS significantly increasing MyD88 mRNA (*p* < 0.05). This LPS-induced increase in MyD88 mRNA was not blocked by NBI-35965 (**Figure 6C**). LPS treatment also significantly increased the PVN CRF mRNA level [one-way ANOVA: *F*_(2,9)_ = 4.332, *p* = 0.048], and this increase was not blocked by NBI-35965 (**Figure 6D**). By contrast, the LPS-induced significant increase (*p* < 0.01) in the IL-1β level [one-way ANOVA: *F*_(2,15)_ = 8.634, *p* = 0.003] was prevented by NBI-35965 (*p* < 0.01; **Figure 6E**). Similarly, an LPS-induced increase in TNF-α levels [one-way ANOVA: *F*_(2,11)_ = 5.46, *p* = 0.023] was also prevented by NBI-35965 pretreatment (**Figure 6F**).

## Discussion

In this study, we examined the influence of TLR4 signaling on MS-induced visceral hypersensitivity and pain in mice. Our data indicated that MS was associated with an increase in visceral hypersensitivity, microglial TLR4, CRF, CRFR1, and inflammatory factors IL-1β and TNF-α protein and/or mRNA expression in *Tlr4*^+/+^ mice. By contrast, MS did not alter visceral hypersensitivity, CRF, CRFR1, IL-1β and TNF-α protein and/or mRNA expression in *Tlr4*^-/-^ mice. Thus, our studies revealed for the first time that TLR4 signaling and neuroinflammation in the PVN are critical for MS-induced adult visceral hypersensitivity and pain.

The present study confirmed that neonatal MS induces visceral hypersensitivity and pain in adult mice. In rodents, MS is a well-characterized model of early life stress that has been used to model depression, anxiety, and IBS. Visceral hypersensitivity is considered a major factor inducing IBS (Wouters et al., 2012). Animals subjected to MS also display increased sensitivity to CRD (Coutinho et al., 2002). Visceral hypersensitivity, a common symptom with a significant impact on the quality of life for patients with IBS, lacks efficient therapies (Chey et al., 2015). Even though the pathogenesis of visceral hypersensitivity is not fully understood and is likely multifactorial, stress or adverse early life events are potential contributors (Al-Chaer et al., 2000; Zhou et al., 2010). We found that neonatal MS induced visceral hypersensitivity, as demonstrated by an increase in AWR scores and a decrease in pain threshold in *Tlr4*^+/+^ mice but not in *Tlr4*^-/-^ mice. MS affects the trajectory of neural development and alters phenotypes during adulthood (Garner et al., 2007; Rentesi et al., 2010; Hulshof et al., 2011). Thus, the results of the present study are consistent with those from earlier studies and extend our understanding to indicate that TLR4-mediated neuroinflammation in the PVN is critical for early life stress-induced visceral hypersensitivity and pain.

The hypothalamic PVN integrates stress, nociception, gastrointestinal function by the HPA axis and autonomic nerve system (Ferguson et al., 2008). We previously demonstrated that the hypothalamic PVN is critical for the pathogenesis of visceral hypersensitivity and pain precipitation in an IBS-like rat model developed by CRD. We showed that visceral hypersensitivity and pain induced by neonatal CRD is associated with an increase in the expression of PVN c-Fos. The PVN collects multiple inputs and generates an integrated autonomic output for pain and analgesia regulation. Consistent with our previous results and the role of the PVN, the present study found that MS was associated with an increase in the expression of both the TLR4 protein and mRNA in the PVN of *Tlr4*^+/+^ mice, whereas levels of TLR4 and MyD88 expression in *Tlr4*^-/-^ mice were significantly decreased.

The increase in the release of IL-1β and TNF-α proteins through the TLR4/MyD88/NF-κB signaling pathway in microglia induces neonatal stress-induced visceral hypersensitivity and pain (Chen et al., 2015). TLR4 is predominantly expressed in microglia with sparse expression in astrocytes and neurons (Lee et al., 2013). It is well-established that TLR4 signaling modulates microglial activation and pathological pain occurrence (Tanga et al., 2005; Lee et al., 2013). Consistent with this, we demonstrated here the co-labeling of TLR4 and Iba-1, a microglial marker. Microglia is the resident macrophage in the brain that detects external information and release a large number of inflammatory factors (Tang, 2014), We previously reported that visceral hypersensitivity was involved in microglial activation, IL-1β and TNF-α accumulation in the PVN (Chen et al., 2015). Consistent with these data, the present study determined that visceral hypersensitivity was involved in an increase in PVN IL-1β and TNF-α protein levels. Glia-derived proinflammatory cytokines are mediators of hyperalgesia (Sommer, 2003; Milligan and Watkins, 2009). It has been posited that inflammatory factors can target PVN CRF neurons to induce visceral hypersensitivity. Our current data indicated that neonatal MS increased the sensitivity of microglial cells, suggesting a potential mechanism for the microglial modulation of visceral hypersensitivity and pain in adulthood. Microglial TLR4 signaling may engage in the onset and/or maintenance of visceral hypersensitivity/hypernociception induced by MS. Inflammatory factors induced by TLR4 activation can act detrimentally or beneficially within the surrounding cells to modulate visceral sensitivity and pain (Kettenmann et al., 2011; Eyo and Wu, 2013). We found that neonatal MS induced an increase in the levels of IL-1β and TNF-α, but not IL-6, in the PVN of adult mice. The downstream targets of these inflammatory factors will require additional study.

Early life stress can alter the developmental trajectory of neuroendocrine system, especially of the HPA axis, and potentiates vulnerability to subsequent stressor and development of pain in adult (Van den Bergh et al., 2008; Green et al., 2011; Amath et al., 2012; Wouters et al., 2012). CRF, originated from neuroendocrine parvocellular neurons of the hypothalamic PVN, regulates basal and stress-induced HPA activation, neuroendocrine, autonomic, immunologic, behavioral and visceral responses to stressor (Kusek et al., 2013). Stress increases HPA axis activity and triggers the secretion of CRF from the hypothalamus. CRF acts at both the central and peripheral targets to regulate gastrointestinal motility (Trimble et al., 2007). Exogenous CRF increases rectal sensitivity (Lembo et al., 1996), and chronic stress enhances parvocellular CRF mRNA expression in the PVN (Makino et al., 1995; Flak et al., 2009). We previously demonstrated that rats experiencing visceral hypersensitivity show an upregulation of CRF neuronal activation as well as an increase in CRF mRNA and protein expression in the PVN and that genetic suppression of CRF expression in the PVN prevented early life stress-induced visceral hypersensitivity (Zhang et al., 2016a). The results of our present study indicated that in animals that did not experience MS, there was no difference in the number of CRF^+^ cells and CRFR1^+^ cells or in the level of CRF mRNA between *Tlr4*^+/+^ and *Tlr4*^-/-^ mice. However, neonatal MS induced a significant increase in the number of CRF^+^ cells and CRFR1^+^ cells and in the level of CRF mRNA in only *Tlr4*^+/+^ mice, not *Tlr4*^-/-^ mice. Consistent with our data, CRFR1 antagonism has been shown to inhibit stress-induced colonic hypersensitivity (Greenwood-Van Meerveld et al., 2005), and CRFR1-deficient mice show decreased anxiety and colonic sensitivity to CRD (Trimble et al., 2007). Together with these data, our results suggest that both CRF and CRFR1 participate in neonatal MS-induced visceral hypersensitivity.

Our data revealed that neonatal MS induced an increase in the protein levels of IL-1β and TNF-α, but not IL-6 in the PVN. Microglia-to-neuron signaling and neuroinflammation have recently received extensive attention in visceral pain perception (Watkins et al., 2001; Tsuda et al., 2005). Microglia-to-neuron signaling is essential for chronic pain hypersensitivity (Sorge et al., 2015). Besides functioning as macrophages in the central nervous system, microglia can also detect environmental information and release many cytokines that can act detrimentally or beneficially on the neighboring cells (Kettenmann et al., 2011; Eyo and Wu, 2013). Additionally, peripheral sensory afferents can provoke microglia to release pro-inflammatory cytokines to precipitate neuropathic pain by acting at nearby neurons (Watkins et al., 2001; Tsuda et al., 2005). Inflammatory factors may serve as potential bidirectional communicators between microglia and neurons for pain precipitation. Regarding the involvement of microglial TLR4 signaling and PVN in visceral hypersensitivity, we posit that the microglial TLR4/MyD88 signaling pathway modulates neonatal MS-induced visceral hypersensitivity via CRF neurons in the PVN. This proposed mechanism provides novel insights for understanding the neuronal and molecular basis of the development and precipitation of visceral hypersensitivity, and may facilitate development of therapeutic approaches in IBS.

To further verify the involvement of TLR4 signaling in the PVN on neonatal MS-induced visceral hypersensitivity, we infused LPS, a TLR4 agonist, locally into the LV. LPS induced visceral sensitivity, which could be blocked by NBI-35965, a TLR4 antagonist. Interestingly, LPS in the LV increased the mRNA expression of TLR4, MyD88, and CRF. Moreover, IL-1β and TNF-α were also elevated by the LPS treatment. These data suggest a causal relationship for TLR4 activation and the expression of CRF and the inflammatory factors TNF-α and IL-1β.

Stress is present across the life and affects the individual development and survival. Besides TLR4 signaling, early life stress can alter oxidative stress and kynurenine metabolism with a decrease in neuroprotective ratio which may underlie the subsequent behavioral and cognitive deficits (Moller et al., 2013). Tissue can be damaged by the accumulation of reactive oxygen species (ROS) and reactive nitrogen species (RNS). To protect organs, ROS/RNS are scavenged via antioxidant defense systems, including endogenous antioxidants (glutathione) and membrane-protecting enzymes, e.g., superoxide dismutase (SOD), catalase (CAT), and glutathione peroxidase (GSH-Px). Oxidative stress, defined as an unbalance between the oxidant generation and the antioxidant response, can impair neuronal development and elict neurological and psychiatric disorders. Early life stressful events, e.g., early MS, can lead to increased oxidative stress in the CNS and enhance the risk to develop psychiatric diseases such as anxiety, depression, drug abuse or psychosis (Schiavone et al., 2015). Various stressors may disrupt the redox homeostasis of tissues by activating stressor-specific pathways and provoke specific responses (Filipovic et al., 2017).

Maternal separation can disrupt neuronal development, causing increased corticosterone sera levels, decreased CD8+ T cell percentage, and elevated spleen T cell CD4/CD8 ratio (Roque et al., 2014). MS before postnatal day 14 augmented antioxidant enzyme activities and suppressed lipid peroxidation in infant rat brain. However, maternal deprivation reduced enzyme activities and increased lipid peroxidation after postnatal day 14 (Uysal et al., 2005). The difference may indicate a critical period of development in which early life stress events strongly modulate mood behavior, immune, and endocrine systems. The MS related oxidative responses in PVN has not been reported. It will be interesting to illuminate the oxidative response in PVN to MS and its influence on visceral pain.

Free radicals and antioxidants are involved in visceral pain. Vaculin et al. (2010) found that visceral pain induced by CRD in adult rats was associated with an increase in free radical scavenging enzymes [glutathione peroxidase (GPx) and SOD] levels in the liver and blood, and a decrease in the brain. TLR4 pathways are crosslink to oxidative stress. Oxidative stress increases surface TLR4 expression (Tawadros et al., 2015). TLR4 senses oxidative stress mediated by the oxidation of phospholipids (Mancek-Keber et al., 2015). Increased activation of TLR4 can lead to the induction of oxidative stress. After TLR4 triggers NF-κB activation, inflammatory factors, such as IL-6 and TNF-α, are secreted. These inflammatory factors accelerate the inflammatory response by reducing SOD activity and increasing malondialdehyde (MDA) production (Deng et al., 2016). Brain may present region-differential susceptibility to oxidative stress following MS (Filipovic et al., 2017). Defining the sources of oxidative stress resulted from early life stress might open new beneficial insights in therapeutic approaches to these mental disorders.

Neonatal MS may alter neuronal plasticity, function, and communication, and increase susceptibility to pain precipitation and later-life psychopathology (Van den Bergh et al., 2008; Green et al., 2011; Amath et al., 2012). In this study, we demonstrated that MS-induced visceral hypersensitivity is associated with an increase in TLR4 signaling in the PVN. Moreover, the expression levels of CRF and CRFR1 are elevated in the PVN. Together, these data indicate that an increase in TLR4 signaling in the PVN is associated with the precipitation of visceral hypersensitivity. Further studies will be necessary to explore the communication between TLR4 signaling and the CRF pathway on MS-induced visceral hypersensitivity.

## Author Contributions

Y-MZ and GZ conceived the idea, designed this experiment, prepared and submitted this manuscript. H-LT, LD, N-NJ, B-BC, and RH conducted this experiment and ran data analysis.

## Conflict of Interest Statement

The authors declare that the research was conducted in the absence of any commercial or financial relationships that could be construed as a potential conflict of interest.
