# Supplementary material for: Toll-Like Receptor 4 in Paraventricular Nucleus Mediates Visceral Hypersensitivity Induced by Maternal Separation
Source: Front Pharmacol. 2017 May 29;8:309. doi: 10.3389/fphar.2017.00309 (PMC5447361; doi:10.3389/fphar.2017.00309)
Supplement: Supplementary file 1 [file Data_Sheet_1.DOCX]

**Supplementary Information**

**Materials and Methods**

*Isofluorane anesthesia*

C57BL mice received isoflurane to induce anesthesia for 15 min (oxygen, 1.0 L/min; isofluorane, 3% induction and 1% maintenance) using an Isofluorane Vaporizer (RWD510, RWD Life Science Co., Ltd., China). The control mice received the same treatment expect isoflurane exposure. Behavioral tests were conducting 2 h after mice waked from isofluorane anesthesia.

*Body temperature*

Body-core temperature was measured in the rectum by inserting a 1.0 cm vinyl-jacketed thermoprobe connected with a digital thermometer. The thermoprobe stayed in the rectum for 1-2 min until a stable readout. Temperature was recorded before and 2 h after isofluorane anesthesia.

*Locomotor activity*

The mouse was placed in the center of a white opaque arena (30 cm by 30 cm by 37.5 cm tall) and tracked via an overhead video camera interfaced with behavioral tracking software EthoVision XT 5.1 (Noldus Information Technology, The Netherlands). Distance traveled (the recorded movement of the mouse’s center point in m over the 5-min duration of the trial) and velocity were calculated.

*Grasping Test**ing*

The grasping testing was conducted with a dynamometer (YLS-13A, Jinan Yiyan Technology and Development Co., Ltd., China). In brief,  after habituated to the recording environment, mice  was  gently  placed  on  the  grid  of  the  dynamometer and pulled by their tails in the opposite direction. The maximum grip strength exerted before losing grip was recorded. Each mouse was tested for three times with an interval time of 30 s, and the grip strength was indicated by the mean of the three measurements.

*Visceral pain threshold*

Mice were fasted for 18 h with water *ad libitum* before testing. After habituation to a small Lucite cubicles (20 cm × 20 cm × 9 cm)15 min, a custom-designed balloon (1 cm in diameter and 2 cm in length), connected to a syringe for inflating the balloon via a tube, was inserted into the colorectal intestine 0.5 cm above the anus under isoflurane anesthesia. The distension pain threshold was defined by a stimulus intensity that evoked a visible contraction of the abdominal wall. During pain threshold testing, colorectal distension (CRD) was applied in increments of 10 mmHg starting at 10 mmHg for a duration of 20 s followed by a 4-min break. To achieve an accurate measurement, the distension at each condition was performed in triplicate to achieve an average for further analysis.

Results

There was no difference in body core temperature (Fig. S1A; *t*(8) = 0.286, *p* = 0.782; n = 5 in each group), distance traveled (Fig. S1B; *t*(8) = 1.032, *p* = 0.332; n = 5 in each group), velocity (Fig. S1C; *t*(8) = 1.014, *p* = 0.34; n = 5 in each group), grasping power (Fig. S1D; *t*(8) = 0.011, *p* = 0.991; n = 5 in each group), and visceral pain threshold (Fig. S1E; *t*(4) = 0.043, *p* = 0.968; n = 3 in each group) between the control group and isofluorane-exposed group.





Figure S1. Effect of isofluorane on mouse behaviors. Mice received isoflurane to induce anesthesia for 15 min. Behavioral tests were conducting 2 h after mice waked from isofluorane anesthesia. There was no difference in body core temperature (A; *t*(8) = 0.286, *p* = 0.782; n = 5), distance traveled (B; *t*(8) = 1.032, *p* = 0.332; n = 5), velocity (C; *t*(8) = 1.014, *p* = 0.34; n = 5), grasping power (D; *t*(8) = 0.011, *p* = 0.991; n = 5), and visceral pain threshold (E; *t*(4) = 0.043, *p* = 0.968; n = 3) between the control group and isofluorane-exposed group. Data are expressed as mean ± SEM.
